# Transactions of the Provincial Medical and Surgical Association. Vol. III. 1835 Mr. James on the Causes of Good or Bad Stumps

**Published:** 1835-07-01

**Authors:** 


### The Transactions of the Provincial Medical and Surgical Association. Instituted 1832. Volume III. 8vo. pp. 472. London and Worcester, 1835.

This volume has arrived too late to enable us to lay a complete account of its contents before our readers. It consists of an eloquent anniversary address from Dr. Conolly—of a Report on the Chemistry of the Blood, and on the present state of our Knowledge of Anatomy—of nine insulated essays or cases—of three Hospital Reports—a Biographical Sketch of the late Dr. Jackson, Inspecter of Military Hospitals—of an Article of Medical Jurisprudence on the Bristol Case of Poisoning—and of a Report from Van Dieman's Land. Such are the contents of the volume before us, and all we can do, upon this occasion, will be to select one of the shorter articles for notice here, deferring the remainder until our next Number. The portion devoted to hospital reports will be fully reviewed in our Clinical Department. The paper to which we shall now allude is from the pen of Mr. James, surgeon of the Devon and Exeter Hospital, and already known in a favourable manner to the profession.

### Observations on some of the Causes which influence the Formation of Good or Bad Stumps, in certain Cases of Amputation of the Thigh. By J. H. James, Esq. Surgeon to the Devon and Exeter Hospital.

Mr. James remarks on the varieties of opinion that prevail, with respect to the best method of operating. But it would be difficult to discover a point in surgery or medicine on which varieties of opinion do not prevail. We may, therefore, pass them over, and content ourselves with stating Mr. James's observations. They are limited to the operation by the circular incision.

The surgeon's object is to save sufficient muscle to cover the bone, and sufficient integument to cover both. Bad stumps may result from an original defect in the operation, or from something wrong in the subsequent processes.

To avoid defects in the operation, there are many circumstances connected with the condition of the limb, that the surgeon should carefully consider. Supposing the limb to be healthy, or nearly so, Mr. James would draw attention to these general principles.

"The muscles ought to meet over the bone, and the integuments over the muscles; the object is to know how this may be best accomplished, indeed, why not always attained. The necessary quantity of integuments, as Mr. Hey has stated, may be ascertained by admeasurement (a practised eye will rarely require it); but with respect to the other constituents, the muscle and bone, the quantity is not so easily determined. The proportion of the former saved may vary, both on account of its natural bulk at the part, which is less as we approach the knee, and also on account of the degree of contractility of the loose muscles, which will differ according to their condition at the time, and will be more or less affected by the application of a tourniquet, or other circular band. The proportionate diameter of the bone to other parts in the section, it is important to observe, will also differ materially: thus, its diameter considerably increases towards the knee, while that of the muscular flesh diminishes in the same ratio. The converse is true as we approach the middle of the thigh; and hence it sometimes happens with regard to amputation in the latter situation, that while the small extremity of bone is well covered with muscle, the surgeon may not have allowed sufficiently for the diameter of the limb at this point, so much exceeding that at the usual place, and if so, the integuments will be scanty, an evil always obviated by attention. It is not so if the section of the bone be made too near the knee; an error to which the directions of some approved authors would lead, or if the bone, at the lower part of the limb, be enlarged, or the muscles wasted by disease, as hereafter particularly stated; for in these various cases, although we may easily preserve an ample covering of integument, we shall fail in getting a cushion of muscle for the broad bone, unless we clear up the muscle from it to a considerable distance before sawing, a proceeding from which we shall reap this further advantage—that the section of bone will be made where the shaft is smaller." 221.

Mr. James considers next the deviations from a healthy condition of the limb, which are capable of affecting the formation of a good stump.

*First.* The skin may be loose and redundant. This is commonly the case in old people. Two evils result from saving an unnecessary quantity of skin; first, the facility thereby presented to the collection of matter: secondly, that, as in closing the stump we act upon the muscles by drawing down the integuments, we are deprived of this advantage when the latter are superabundant, and the muscles consequently will not meet over the bone, or if we persist in thus forcing them down, the integuments will form a loose pouch over its extremities. Mr. James conceives that the plan which has been recommended by some authors, of dividing the integuments (previously drawn up), together with the loose muscles, by the first incision, is well adapted to these cases.

*Secondly.* When there is an excess of adipose substance, this is not unfrequently so redundant and so hard, as to prevent the accurate closure of the wound. During the process of suppuration, this will often disappear; but if its existence is recognized prior to the operation, Mr. James recommends the surgeon to incline the edge of the knife a little upwards in making the first incision.

"While upon this point, I may remark, that we are sometimes compelled to operate upon limbs in an anasarcous state; should this be the case in any considerable degree, it is not a bad plan to roll the limb firmly before the operation, but even then, it is hardly possible completely to empty the cells, and there will be a drain of serum from the edges at first, which, however, if matters go on well, will soon subside. In these cases it is not safe to trust to the tourniquet." 223.

*Thirdly.* "The muscular flesh of the limb may be much changed in various ways: it may be simply emaciated; or it may be swollen and agglutinated, either from acute or chronic inflammation. In the first case, a good stump will hardly be formed, unless the bone is cleared high up; in the second, from want of contractility of the muscles, a similar proceeding will be essential; but it is to another degeneration that I now particularly solicit attention, in which, from long disuse, (the consequence of old-established disease in the knee or leg) scarcely any trace of muscle is left in the lower part of the limb; the fibre has been for the most part absorbed, and either we find mere fat in large quantity, or intermixed with a little fibre; or, what is not uncommon, the muscle converted into a sort of firm gristly semiadipose matter, perfectly incapable of contracting under the knife. Now, in such cases as these, I cannot but suspect that either of the methods practised by M. Dupuytren, or Sir C. Bell, or those of many other surgeons would fail; they are all based upon the principle that the muscles contract as they are divided, which is not at all the case here; the only mode of forming a good stump, I apprehend in these cases, is by clearing and sawing the bone high." 223.

The muscles may not have degenerated, but their relative contractility may display great differences. For instance, the knee may have been for some time retained in a bent position. The flexors having already contracted, will contract little more upon division. But the extensor muscles, which have been in the same proportion on the stretch, will retract much more; if, therefore, both are divided in the same plane, the latter will be deficient, the former projecting. The separate division, at different heights, of these two classes of muscle, will enable us to overcome this defect; and the position of the limb, after the operation, may be so regulated as to give an advantage to the defective portion.

*Fourthly.* The state of the bone may be altered. When we amputate for necrosis of the thigh, the bone, periosteum, and adjacent cellular tissue may be greatly thickened, and the neighbouring muscles may be much agglutinated. Integument is the thing to be saved in such a case. If this can be effected without detaching it from the subjacent muscles, so much the better; if not, the knife must be employed.

Mr. James observes that a little care and consideration will often enable us to ascertain the presence of any of the preceding changes, before we perform the amputation. The state of the integuments as to looseness, or the reverse; as to thickness, or anasarca, may easily be determined by pinching them up, or rolling them, or other mode of examination. The thickness of the *bone* may often readily be felt, and in cases of necrosis it is very important it should be. The size of the *muscular portion* may be presumed, from a comparison of the limb with the other; its condition, from the degree of rigidity, or otherwise, under the integuments; from the power of distinguishing the bone beneath it, more or less readily; from the nature and duration of the disease, the position of the limb, and other concurrent circumstances. In fine, when we are likely to meet with bone of large relative diameter, we must take care to save sufficient integument; and, if muscle is deficient in quantity or contractility, it is important to clear it up high from the bone.

Mr. James next dwells on the progress of stumps after amputation. We extract the following not uninteresting observations.

"There can be no doubt that a high degree of inflammation, occurring after amputation, will, directly or indirectly, ruin any stump; partly by the disorganization it produces; partly by irritating the muscles to constant contraction; partly by compelling us to give up the circular roller, upon which so much depends: however, these points of surgery have been so fully handled, that it is unnecessary for me to dwell upon them. The evil consequences of redundancy or deficiency of integument, have also been fully pointed out; but in connexion with this subject, it may not be amiss to observe, that there seems to be, in all cases of amputation, a disposition in the skin to relax or contract; the latter, if irritated; the former, if soothed. By soothing, a good covering is sometimes obtained, although there may have been a deficiency in the first instance; while, by irritating, a good stump is often spoilt. When this relaxing process does not take place kindly, it may be advantageously solicited by fomentations or poultices to the face of the stump, often without giving up the steady pressure of the roller above.

The condition of the muscular flesh will also produce a great influence on the progress of the stump; if that has been merely and simply emaciated, it is not at all uncommon to see it plump up after the operation, and, in consequence, a better cushion is, in many cases, afforded to cover the bone, than might have been at first expected; but, on the other hand, it not unfrequently happens that the integuments, which at the time of the operation met easily, become separated by a considerable interval, in consequence of this enlargement of the muscular flesh. If, in these cases, both muscle and skin are early united over the bone, the stump will be much better than it promised to be at first, for the muscle will fill out, and the skin elongate; but if the union is long protracted, the edges of the integuments will be separated by the enlargement of the muscular flesh, and the stump prove defective. The reverse of this is the case when, before amputation, the size of the muscular portion has been augmented, either by recent inflammation, or where it consists largely of that semi-adipose structure which has just been described, both of which will waste during the suppurative process. Should the integuments have been at all deficient at the time of the operation, they will become slack and easy; and in this respect an important improvement will take place, provided it is only the parts subjacent to the integuments which are constituted by false flesh, if I may so express myself; but if the integuments are also anasarcous, or loaded with gelatinous effusion, they will likewise waste. In all these cases a perfectly plump stump will not be formed, unless the bone be cleared high up.

I have but one more remark to make on this subject, namely, that when the soft parts are deficient, and the bone prominent on the face of the stump, forcible attempts are often made to bring the integuments together by adhesive straps; these endeavours commonly fail, and are, indeed, mischievous, for if the straps are brought over the surface of the bone, then they bind down this thin and irritable covering upon a hard surface, which is sure to indispose them to unite or do well; and if applied at the sides, although they may bring the edges together, yet they will force the soft parts still more back from the bone. The only remedy for such stumps, is to be found in good rolling, and proper position; or, these failing, in sawing off the bone higher." 229.

Mr. James concludes by drawing a sort of analogy between the wound produced by amputation, and that occasioned by a compound fracture. But on this we need not dwell. We may confine ourselves to one practical remark:— That surgeons not unfrequently persist in the attempt at union by the first intention too earnestly and too long. If it does not succeed at once, the straps should be partially cut at all events, so as to prevent the confinement of matter, at the same time that the bandages are left sufficiently undisturbed to obviate the danger of what union there may be, giving way.

Mr. James believes that the outer surface of the bone, being inflamed, not unfrequently furnishes a deposit of ossific matter, in which the nerves may be engaged, and into which they may be pressed by the bandages and straps. Pain, heat, and *solid* swelling are the signs of this condition; leeches, evaporating lotions or poultices, and blisters are the treatment.

We recommend the preceding remarks to the surgical portion of our readers.

